# Ecofriendly green synthesis and characterization of silver zinc oxide nanocomposite using the aqueous leaf extract of Rumex Crispus: Evaluation of its antimicrobial and antioxidant activity

**DOI:** 10.1016/j.heliyon.2023.e16063

**Published:** 2023-05-05

**Authors:** Yigezu Mekonnen Bayisa, Tafere Aga Bullo, Ketema Beyecha Hundie, Desalegn Abdissa Akuma, Defar Getahun Gizachew, Mohammed Seid Bultum

**Affiliations:** School of Chemical Engineering, Jimma Institute of Technology, Jimma University, P.O. Box 378, Jimma, Ethiopia

**Keywords:** Silver zinc oxide nanocomposite, *Rumex crispus* extract, Definitive screen design, Antimicrobial activity, Antioxidant activity

## Abstract

The hydrothermal approach is used in the current study to create an environmentally friendly silver zinc oxide nanocomposite utilizing an aqueous leaf extract of Rumex Crispus. The photochemical components of Rumex Crispus, a synthetic nanocomposite with antioxidant and antibacterial activity, were also assessed. The Response Surface Methodology of Definitive Screen Design (DSD) was used to examine and optimize the effects of four independent variables on the amount of green synthesized silver zinc oxide nanocomposite in Rumex Crispus extract. According to the experimental findings, the green synthesized silver zinc oxide nanocomposite's maximum 1.89 absorbance intensity was achieved at a reaction temperature of 60 °C, a concentration of silver nitrate salt of 100 mM, a pH value of 11, and a reaction period of 3 h. The synthesized nanocomposite was characterized using Fourier-transform infrared, UV, X-ray, UV–vis, Dynamic Light Scattering, thermogravimetric analysis, and differential thermal analysis to determine its functional group, structure, bandgap energy, size distribution, a mass of loss, and energy gain or loss, respectively. The minimum lethal doses for the gram-positive, gram-negative, and fungal strains were 1.25, 0.625, and 2.5 g ml^−1^ respectively. The 1-1-diphenyl-2-picryl hydrazyl (DPPH) which was used to measure antioxidant activity is scavenged by Ag–ZnO nanocomposites, and the IC50 value of a Rumex Crispus extract is 29.31 g ml-1 IC_50_ value is.29.31 μg ml^–^^1^. Their findings show that Rumex Crispus extract-derived synthetic silver zinc oxide nanocomposite is a promising alternative against both Gram-positive and Gram-negative bacterial strains and fungal strains, as well as a prospective choice for antioxidants under the given conditions.

## 1 Introduction

Since ancient times, many regions of Ethiopia have produced numerous treatments for various illnesses using various plant components through traditional techniques [1]. Several scholars are currently using these plant parts in conventional ways to find new treatments for a variety of diseases [2,3]. According to theories and works examined by academics, the majority of medications utilized by numerous people worldwide were first derived from natural plants [4]. However, the cost of many current medications, including those used to treat antibiotic resistance, renders them worthless in many areas [5,6]. Nowadays, nanoparticle production and its applications have become a significant technological advancement with numerous uses in a variety of industries, including catalysis, molecular sensing, environmental remediation, and medicine [7,8]. Associated with bulk materials, nanomaterials exhibit entirely new or improved properties due to specific properties such as size, distribution, and morphology [9,10].

The biosynthesis of nanomaterials using plant extracts and microbes has garnered a lot of attention from researchers in recent years [11]. This makes nanocomposites suitable for medical, photocatalysis, bio-imaging, and bio-sensor applications [12]. In addition, the biological process makes it cheaper, greener, and superior to chemical and physical processes [13]. In the area of metal oxide nanoparticles, including ZnO, CuO/C, Ag, Au, Pd, and Cd, have proven to be particularly beneficial for medicinal applications. Among others, ZnO is One of the most utilized biocompatible transition metal oxide semiconductors of the II-VI semiconductor group among other semiconductors [2,14]. ZnO nanoparticles are thought to be safe since they are biocompatible, may last for several hours inside the body, have high chemical stability, low cost, large surface area, and have a variety of biological uses due to their quick dissociation and absorption [15,16].

However, ZnO has a low photocatalytic degradation rate and its activity can be enhanced by incorporating metal ions because charge carriers recombine quickly and there is relatively little charge separation [17]. Additionally, the photocatalytic activity of the nanostructured ZnO with a broad bandgap of 3.37 eV and binding energy of 60 meV is restricted to the UV area and is inactive when exposed to visible light [15,18]. Therefore, it is important to create nanocomposites with other materials that contain metal ions, such as silver, to enhance the photocatalytic capacity, antioxidant, and antibacterial activity (Ag) and iron (Fe) ions [[19], [20], [21]]. It may increase the photocatalytic activity of particles since nanoscale Ag^2+^ alone has antimicrobial properties, but it can also be a highly intriguing and helpful addition to ZnO nanocomposites [22,23]. Synthesis of metal oxide nanomaterials from *Medicago sativa, Ulva, Achillea biverstani, Moringa oleifera, Calendula officinalis, Peganum harmala, Green tea, Pistacia Atlantica, Olive, Aloe vera, and Coriandrum sativum* was investigated by different scholars [24]. *Rumex crispus*, commonly known as ‘cur dock’ because of its wavy, curled leaf, is a perennial plant native to most African regions, including Ethiopia, and often used in traditional medicine as a useful substitute. It is useful as a treatment for sexually transmitted diseases and is used interchangeably with blood purifiers [25].

In this study, *Rumex Crispus* leaf extracts were considered for the synthesis of ZnO–Ag nanocomposites that will be utilized for the green synthesis of nanocomposites and evaluation of antimicrobials and antioxidant activities.

## 2 Materials and methods

### 2.1 Materials and reagents

The Silver nitrate (AgNO_3,_ 99%), Zinc acetate hexahydrate (Zn(CH_3_OO)_2_.2H_2_O, 98%, extra pure), Muller-Hinton agar, Ethanol (96%), Potassium hydroxide (extra pure) and mercury produced by Loba Chemicals Pvt. Ltd in India was purchased from Atomic Educational Materials Supply PLC in Addis Ababa, Ethiopia. *Rumex Crispus* was acquired from Jimma, Southwestern Oromia, Ethiopia. All the other chemicals were analytical reagent grade and bought from Chem-Supply Kirkos Ltd. in Addis Ababa, Ethiopia. All reagents used in this study were pure analytical grade.

### 2.2 Preparation of *Rumex Crispus* leaf extract

Freshly obtained Rumex Crispus leaf weighing 10 g were cleaned, dried, and divided into smaller pieces. In a beaker, 100 ml of distilled water was combined with the powder. The solution was transferred to a magnetic hotplate equipped with the beaker and was held at a temperature of roughly 80 °C for 3 h. The Rumex crispus extract was filtered after 3 h using folded filter paper and kept in a closed jar for later use.

### 2.3 Synthesis of Ag–ZnO nanocomposites

Ag–ZnO nanocomposites were made by using the hydrothermal process [26]. Typically, 0.15 g of zinc nitrate hexahydrate (Zn(NO_3_)_2_.6H_2_O) and 0.17 g of potassium hydroxide were dispersed in solutions with an equal mixture of distilled water and mercury ethanol (16 ml). Using a stirrer, zinc nitrate solution was added in a dropwise fashion to the sodium hydroxide solution. The preceding KOH/Zn(NO_3_)_2_.6H_2_O solution was then added, and it was agitated for 5 min. Afterward, silver nitrate solution (1.96 mg in 4 ml of 50% ethanol) and condensed extract of Rumex Crispus was added. The complete samples were moved to the Teflon liner and autoclaved for 12 h at 120 °C in a hot air oven. After cooling to room temperature, the combinations were rinsed with distilled water. The mixes were then centrifuged numerous times after cooling to room temperature and being successively rinsed with distilled water and ethanol. The mixes were then dehydrated at room temperature before being heated to 400 °C for 2 h.

### 2.4 Phytochemical analysis of *Rumex Crispus* leaf extract

The method described in Ref. [1] was used to assess the content of phytochemicals such as alkaloids, flavonoids, saponins, tannins, terpenoids, and phenol in crude Rumex Crispus Leaf Extract.

### 2.5 Characterization of the synthesized Ag–ZnO nanocomposites

Ag–ZnO nanocomposites were made, and their confirmation required the use of a variety of techniques, including XRD analysis, FTIR analysis, TEM evaluation, Duckworth-Lewis-Stern technique (DLS), and UV–Vis diffuse spectroscopy. With the aid of FTIR spectroscopy, the subsurface chemistry of newly produced Ag–ZnO nanocomposites can be investigated. The effectiveness of using KBr for stable samples has been evaluated (powder form of Ag–ZnO Nanocomposite). Using the Zetasizer Nano equipment, dynamic mild Scattering analysis (DLS), a well-known technique, can be used to describe the common particle length distribution of nanocomposites. Using an X-ray diffractometer, the crystalline structure of the biosynthesized Ag–ZnO nanocomposites was studied. Utilizing CuK radiation (= 1.54060 A°) and the DW-XRD-Y7000 instrument, the XRD analysis was performed. Using a wavelength test between 200 and 800 nm, the UV–Vis absorption spectra of the biosynthesized ZnO nanoparticles and Ag–ZnO nanocomposites from the Rumex Crispus extract were examined. The bandgap power of the biosynthesized Ag–ZnO nanocomposites was also detected using the results of the characterization approach.

### 2.6 Antimicrobial activity test

The natural antibacterial properties of the produced Ag–ZnO nanocomposites were investigated according to the methodology described in Ref. [27]. Then the disc diffusion method was used to determine the antimicrobial interest using a suspension of bacteria and fungi dispersed on nutrient agar.

Nutrient Muller Hinton Agar medium was used to keep bacterial cultures whereas Sabouraud Dextrose Agar medium was used for fungal cultures. All bacterial and fungal traces were cultured for 24 h at 37 °C for the strains and for 48 h at 27 °C, respectively. Using the disk-diffusion method, the antibacterial activity of the synthesized Ag–ZnO nanocomposites against the Gram-positive *Staphylococcus aureus* and the Gram-negative *Escherichia coli* was studied. Likewise, the antifungal interest of synthesized nanocomposites towards Candida albicans become similarly tested. Ciprofloxacin and Dimethyl Sulfoxide (DMSO) became used as a control against bacteria and fungi respectively.

Hence, the antimicrobial examination was started with the use of inoculums, wherein two agar plates were placed with nutritional broth that contained 28 mg and was dissolved in 100 ml of distilled water. Microorganisms from different cultures have been swabbed onto the plates containing nutrient agar media. The bacterial cultures that had been added to the nutritional broth had been shaken vigorously for 24 h at a speed of 100 rpm.

One by one, 10 ml of distilled water was used to dissolve 200 mg of a synthesized Ag–ZnO nanocomposite to create solutions containing 20 mg/mL. A sample of Ag–ZnO nanocomposite and an antibiotic disc were placed on separate portions of the two agar plates. Therefore, using a dispenser that dispenses a few discs at a suitable distance apart without undesirable zone overlapping, controls (20 mg/ml) and Ag–ZnO nanocomposites (20 mg/ml) were deposited at the bottom of each agar plate. The inoculation plates had undergone a 24-h period of incubation at 37 °C. By assessing the percentage of inhibition against the test fungus and microbe, the antimicrobial interest was assessed.

### 2.7 Determination of minimum inhibition concentration (MIC)

The minimal inhibitory concentration (MIC) of the ZnO–Ag nanocomposite become tested by way of the Microdilution broth approach described in (Mekonnen and Bultum, 2022). To determine the zones of inhibition, 20 g of Ag–ZnO nanocomposite was dissolved in 1 ml of dimethyl sulfoxide (DMSO) and prepared as a nanocomposite suspension using an ultrasonic tool. Then, 100 l of Ag–ZnO nanocomposite solution and 100 l of Muller-Hinton Broth were administered to all of the property's microplates as the culture medium for microorganisms and fungi, respectively. The produced Ag–ZnO nanocomposite aggregate was periodically diluted in the living medium to the following serial concentrations: 5, 2.5, 1, 25, 0.625, and 0.313 mg/ml. Every test used the investigated microorganism lifestyle medium as the good control and all dilutions of nanocomposite solution with each microorganism lifestyle medium as the bad control. The microplates were then incubated for 48 h at 37 °C. 10 l of each hollow space were cultivated on Sabouraud dextrose agar way of life medium and Muller-Hinton Broth culture medium, and they were incubated at 30 °C for 48 h to determine the minimal lethal awareness (MBC). The plates have been examined for the presence of bacteria or fungi. The minimal lethal concentration (MBC) of fungi or microorganisms was defined as the concentration of nanocomposite solution that inhibited ninety-nine percent of bacterial or fungal cells.

### 2.8 Antioxidant activity test

The 1-1-diphenyl-2-picryl hydrazyl (DPPH) test was used to measure antioxidant activity. A 50 μl of synthesized Ag–ZnO nanocomposites with ascorbic acid were added to a 5 ml methanol solution of DPPH cautiously shaken and put in darkness. After 30 min, the sample absorbance was read at 517 nm using a spectrophotometer. The quantitative estimate of the scavenging capacity of free radicals was determined as equation (1).

Eq. 1 $$I,\%=\left(\frac{A_{blank}-A_{sample}}{A_{blank}}\right)\times 100$$

where *A*_blank_ is the absorbance of the control reaction (containing all reagents except for test compound), and *A*_sample_ is the absorbance of the test compound.

### 2.9 Experimental design for synthesis Ag–ZnO nano composites

Experimental data analysis was done using Design- Expert 13.8.0 software. The experimental design selected for this study is two-level-four-factor response surface methodology of the Definitive screen design (DSD) model for the parameter variable of reaction temperature, concentrations of silver nitrate, pH value, and reaction time [28].

## 3 Result and discussion

### 3.1 Phytochemical analysis of *Rumex Crispus* extract

Table 1 showed that the plant of Rumex Crispus extract became screened for important phytochemicals. The consequences depicted that the Rumex Crispus extract become wealthy in total phenolic, protein, decreasing sugar, flavonoids, saponin, tannins, and terpenoids. Therefore, from the phytochemical screening outcomes, the presence of phenols, flavonoids, and terpenoids permits the plant extract to self-bring together and cap the metallic nanocomposite formed that's used to manipulate morphological shape, lessen and stabilize bulk substances of the host and dopant precursors and their ensuing steel ions [29]. It is proposed that in aqueous plant extracts, the presence of these compounds could be responsible for the bio-reduction of the metal salts into nanocomposites and may play a role in the formation of Ag–ZnO nanocomposites [5].

**Table 1:** Qualitative phytochemical screening of *Rumex Crispus extract*.

Phytochemical test	Name of Detection Test	Inference
Phenols and Tannins	FeCl_3_	+
Steroids	Liebermann	–
Alkaloids	Wagner's	–
Saponins	Frothing	+
Flavonoids	Lead (II) acetate	+
Terpenoids	Salkowski	+
Protein	Biuret	+
Reducing sugar	Molich's	+

### 3.2 Statistical analysis

Each model is significant at a level less than 0.0001 in Table 2, which also lists the significant coefficients of the responses for each model. Given that the two factorials versus linear model was aliased for the respondent responses were extremely significant (p 0.0001), the statistical analyses demonstrate that design models fit the data very well. The Ag–ZnO nanocomposites were synthesized using Rumex Crispus extract, as indicated by the Model F value of 901.1, which suggests the model is important. A, C, B, D, A^2^, B^2^, C^2^, and D^2^ were discovered to have a substantial impact on the creation of Ag–ZnO nanocomposites during the process of synthesis. Ag–ZnO nanocomposites were synthesized using the model, which could properly fit the experimental data, according to the analysis of variance for the lack of fit test (P > 0.05).

**Table 2:** Analysis of variance for Definitive Screen Design model of synthesis of Ag–ZnO nanocomposites.

Sources	Sources Sum of squares	Degree of freedom	Mean of squares	F-value	*P*-value	Remarks
Model	2.30	8	0.2880	901.1	0.0001	significant
A-Temperature	0.6703	1	0.6703	2096.69	0.0001	
B- Concentration of AgNO_3_	0.9554	1	0.9554	2988.60	0.0001	
C-pH	0.2427	1	0.2427	759.29	0.0001	
D-Reaction time	0.0272	1	0.0272	85.23	0.0008	
A^2^	0.0176	1	0.0176	55.15	0.0018	
B^2^	0.3496	1	0.3496	1093.59	0.0001	
C^2^	0.0097	1	0.0097	30.29	0.0053	
D^2^	0.0094	1	0.0094	29.39	0.0056	
**Residual**	0.0013	4	0.0003			
Lack of Fit	2.40	12	0.3143	0.9501	0.5737	not significant

### 3.3 Influence of process parameter on the synthesis of Ag–ZnO nanocomposites

**Fig. 1:**
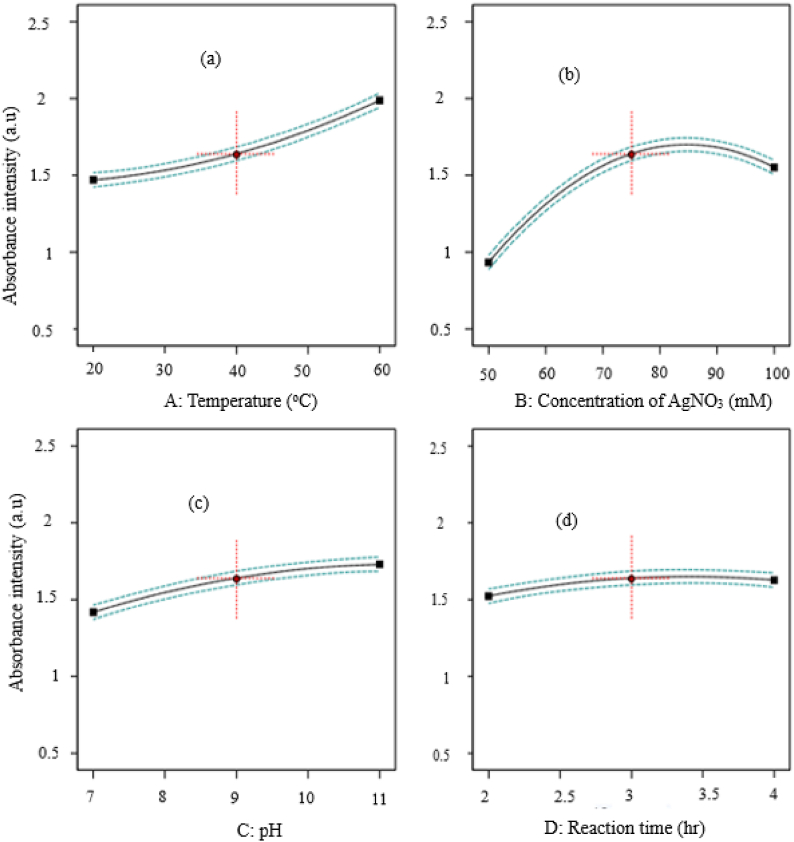
Effects of (a) temperature (b) Concentration of AgNO_3,_ (c) pH value, and (d) reaction time on synthesized absorbance intensity of Ag–ZnO nanocomposites.

As can be seen in Fig. 1(a) to (c), the effects of temperature, AgNO_3_ concentration, pH level, and reaction time on the absorbance intensity of Ag–ZnO nanocomposites were studied. According to the curve in Fig. 1, the absorbance intensity increased noticeably from 1.40 to 1.89 as the temperature increased from 20 to 60 °C (a). Above 40 °C, there has been a modest increase in absorbance intensity, showing that using high temperatures has little impact [30]. The solution's effective temperature is 40 °C with a minor increase in absorbance intensity, according to the results of the Ag–ZnO Nanocomposites absorbance intensity as a function of temperature analysis. Fig. 1(b) illustrates the effect of Ag salt concentration on the absorbance intensity of synthesized Ag–ZnO nanocomposites in addition to the effect of temperature, showing that the minimum absorbance intensity was obtained at 50 mM Ag salt concentration, which is 0.925, and that it significantly increased to 1.65 as the concentration of Ag salt increased to 80 mM [31,32]. Thus, it is evident to say that variations in the concentration of Ag salt and beyond 80 mM caused changes in the absorbance intensity of Ag–ZnO nanocomposites to vary, showing that using the concentration of Ag salt has minimal effect on the absorbance intensity. Additionally, the effects of pH and reaction time was studied since the formation and morphological nanocomposites varies with their values. Here in, the *Rumex crispus* leaf extract was subjected to acidic and slightly basic conditions in order to analyze the development of Ag–ZnO nanocomposites. The *Rumex crispus* leaves extract was 6, but it was increased to 10.9 when metal precursors were added. This might be explained by the fact that some *Rumex crispus* leaves phytocompounds release OH ions when they are oxidized in the presence of metal precursor ions. This suggests that the Rumex crispus extract's low pH prevented the synthesis of nanocomposites in the medium, which may have been caused by the inactivation of phytocompounds necessary for reducing and capping metal precursors [32]. But when the pH was higher (8–10), nanocomposite production was seen since agglomeration results in large-sized nanocomposites. When the pH value and reaction time were changed during the synthesis of Ag–ZnO nanocomposites, as shown in Fig. 1(c) and (d) there was a discernible difference in the absorbance intensity of the Ag–ZnO nanocomposites. As a result, the maximum absorbance intensity is found at pH 9 and a reaction time of 3 h as shown in Fig. 1 (c) and (d).

### 3.4 Characterization of the synthesized Ag–ZnO nanocomposite

**Fig. 2:**
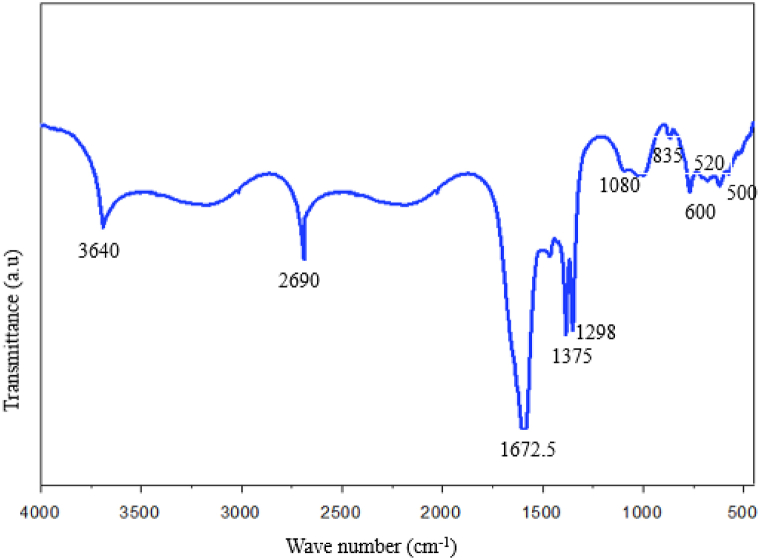
Ftir of ZnO–Ag nanocomposites.

Fig. 2 displays the typical Ag–ZnO nanocomposite and Rumex Crispus extract Fourier Transform Infrared (FTIR) spectrum. The hydroxyl groups, the symmetric C–C stretch, the C–N stretches, and the C–H bonds in the alkane group at 3640, 1625, 1080, and 835 cm^−1^, respectively, were the potential functional groups that were found in nanocomposite [33]. At 1672.5 cm^−1^, a high absorption peak indicated the existence of an amino group and a strong peak at 1375 cm^−1^ showed the OH bent of phenol or tertiary alcohol. A distinct absorption peak at 1298 cm-1 showed the presence of a main or secondary alcohol's OH group. At 1672.5 cm^−1^, a high absorption peak indicated the existence of an amino group and a strong peak at 1375 cm^−1^ showed the OH bent of phenol or tertiary alcohol. A distinct absorption peak at 1298 cm^−1^ showed the presence of a main or secondary alcohol's OH group. The detected absorption peaks at 2690 cm^−1^ was indicate the formation of Ag–ZnO nanocomposites.

The FTIR results revealed that the Ag–ZnO bond vibration in the ZnO composition was associated with the sharp band at around 520 cm^−1^, and the Rumex Crispus extract components on the surface of the Ag–ZnO nanocomposites were associated with the weak bands in the range of 2350 to 1981 cm^−1^ [34]. Hence, the Ag–ZnO nanoparticles are identified in the FTIR spectrum by absorption between 500 and 600 cm^−1^, which further supports the development of Ag–ZnO nanocomposites using Rumex Crispus extract.

**Fig. 3:**
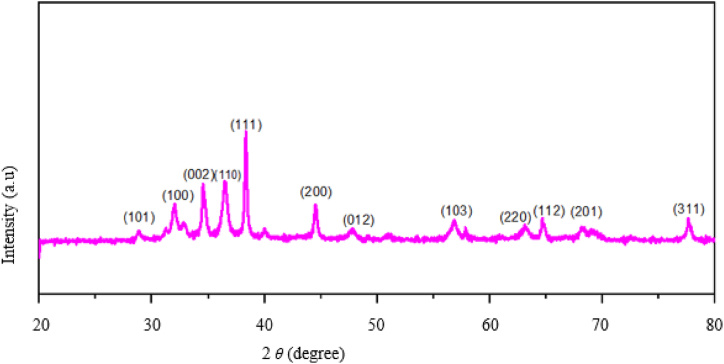
XRD patterns of synthesized Ag–ZnO nanocomposite.

By measuring the powder X-ray diffraction, the XRD pattern of Ag–ZnO nanocomposites made from Rumex Crispus extract was discovered, as shown in Fig. 3. The primary distinctive peaks for Ag–ZnO nanocomposites were located in the crystallographic planes (101), (100), (002), (110), (111), (200), (012), (103), (220), (112), (201), and (311) at 2*θ* values of 29.09°, 32.38°, 34.13°, 37.05°, 38.22°, 44.66°, 47.12°, 57.23°, 62.98°. The face-center cubic silver structure (Ag metallic) and hexagonal wurtzite of zinc oxide (ZnO) were recognized in this XRD pattern. The primary characteristics, which reach their peak at 2 values at 38.22°, 44.66°, 64.77°, and 77.55° and correspond to (111), (200), (112), and (311) respectively, confirm the emergence of silver nanoparticles. The primary characteristic peaks at 2*θ* values 32.38°, 34.13°, 37.05°, 47.12°, 56.23°, 63.77°, and 68.35° that belong to (100), (002), (110), (012), (103), (220), and (201), which are associated to the ZnO phase, can be related to ZnO nanoparticles. According to the XRD study, obvious and distinct phases are forming for both Ag and ZnO, which suggests that a crystalline nanocomposite was created [35]. The average crystal size of the nanomaterials was calculated using Debye Scherer's equation (2).

Eq. 2 $$D=\frac{0.94\lambda }{\beta \;\cos\;\theta }$$

where D is the Crystal size (nm), *λ* is the wavelength of the XRD used, *β* is full-width half maximum (FWHM), and *θ* is Bragg's angle. The X-ray diffractogram of Ag–ZnO nanocomposite materials was analyzed to obtain information about various crystalline aspects and crystallite sizes are approximately 23.44 nm.

**Fig. 4:**
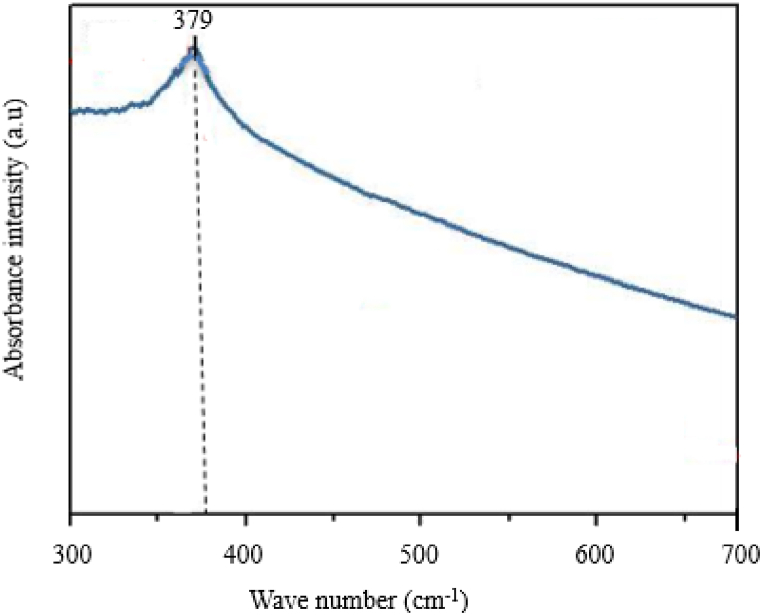
UV visible spectroscopy of synthesized Ag–ZnO nanocomposite.

Fig. 4 depicts the synthesized Ag–ZnO nanocomposite's UV visible spectroscopy. According to Fig. 4, the Ag–ZnO nanocomposite's UV–Vis spectrum was between 335 and 395 nm which displayed a maximum absorption peak at 379 nm. This was demonstrated by a decrease in peak intensity in the ZnO–Ag spectrum, which implied some degree of agglomeration with irregular morphologies. Due to the nanocomposite's addition of Ag impurities, the bandgap for Ag–ZnO was 3.26 eV and Ag^2+^ gradually replaced Zn^2+^ in the materials' matrix, which caused the bandgap to close. Because of their electronegativity and ion radii, these materials' oxygen vacancies systematically increased as a result [36,37].

**Fig. 5:**
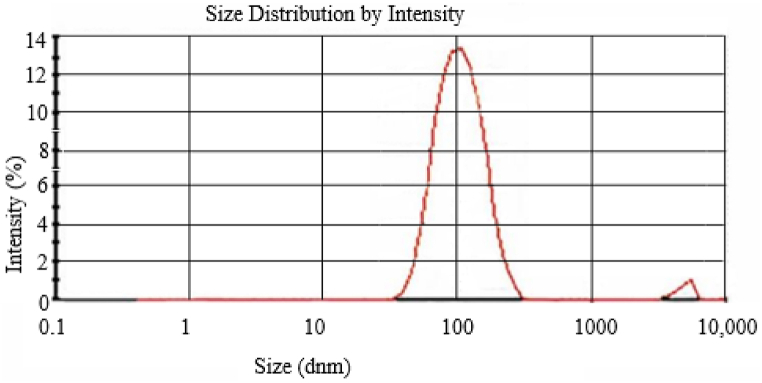
Size distribution of green synthesized *Rumex Crispus* of Ag–ZnO nanocomposite. (For interpretation of the references to colour in this figure legend, the reader is referred to the Web version of this article.)

Thermogravimetric analysis (TGA) and differential thermal analysis (DTA), respectively, were used to determine the mass loss and energy gain or loss of the produced Ag–ZnO nanocomposite. According to Fig. 5, thermogravimetric analysis (TGA) and differential thermal analysis (DTA) reveal that Ag–ZnO nanocomposite lost 36.97% of its mass up to 400 °C but that there was no mass loss from 400 °C to 1000 °C. data suggest that the thermal stability of the greenly produced Ag–ZnO nanocomposite after 400 °C [38].

**Figure:**
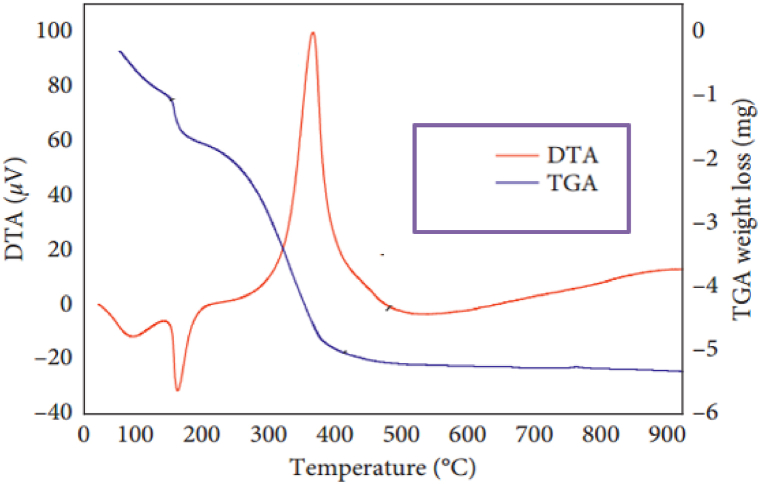

**Fig. 6:**
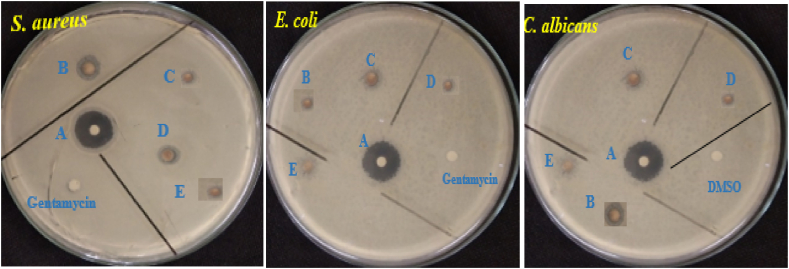
Evaluation of the antibacterial and antifungal activity of Ag–ZnO nanocomposites *Rumex Crispus* extract against *Staphylococcus aureus, Escherichia coli,* and *Candida albicans* A (5 mg/ml), B (2.5 mg/ml), C (1.25 mg/ml), D (0.625 mg/ml), and E (0.3125 mg/ml).

Dynamic Light Scattering (DLS) was used to examine the size distribution of the biosynthesized Ag–ZnO nanocomposite with Rumex Crispus extract, as shown in Fig. 6. With an average particle size diameter of roughly 100 nm and two distinct peaks with intensities of 97.8% and 2.2%, the particle size distribution curve was in the 60 to 140 nm range. It showed that synthetic Ag–ZnO nanocomposite is uniformly sized and homogeneous [39]. The variation in Ag–ZnO nanocomposite sizes could be attributed to the poly-disperse nature of nanoparticles as indicated by the polydispersity index value of 0.251 in Table 3.

**Table 3:** The average particle size of Ag–ZnO nanocomposite.

Peaks	Size (d.nm)	% Intensity	PDI
	ZnO–Ag	ZnO–Ag	ZnO–Ag
Peak 1	100.2	97.8	0.251
Peak 2	4895	2.2	

### 3.5 Antimicrobial activity and minimum inhibition concentration

As shown in Table 4, the antibacterial screening assay of the Ag–ZnO nanocomposite (produced from the extract of Rumex Crispus) was studied in the current study against Gram-positive *Staphylococcus aureus*, Gram-negative *Escherichia coli* bacteria, and Candida albicans fungi. The disk diffusion method was used on these microbial strains to quantify the widths of the inhibition zones for both bacteria and fungi, as shown in Fig. 6. As the concentration of Ag–ZnO nanocomposite extract of Rumex Crispus increased from 0.313 to 5 mg/ml, the inhibition zone diameter for *Escherichia coli*, *Staphylococcus aureus*, and Candida albicans increased from 12.5 ± 0.37 to 22.56 ± 0.12 mm, 11.5 ± 0.22 to 15.5 ± 0.17 mm, and 10.5 ± 0.707 to 17.5 ± 0.207 mm, respectively. The outcomes showed that Rumex Crispus extract had a negative impact on *Staphylococcus aureus* when it was combined with produced Ag–ZnO nanocomposites.

**Table 4:** Zone of inhibition (mm) of Ag–ZnO nanocomposites Rumex Crispus extract.

Zone of Inhibition (mm) (diameter)				
Tested samples	Minimum inhibition Concentration (MIC)	Bacteria strain		Fungi strain
		*Staphylococcus aureus*	*Escherichia coli*	Candida albicans
Ag–ZnO nanocomposites	0.3125 (μg mL^*−*1^)	12.5 ± 0.37	11.5 ± 0.22	10.5 ± 0.707
	0.625 (μg mL^*−*1^)	13.5 ± 0.11	12.89 ± 0.44	11.95 ± 0.52
	1.25 (μg mL^*−*1^)	16 ± 0.31	13.0 ± 0.36	13.25 ± 0.25
	2.5 (μg mL^*−*1^)	19.5 ± 0.17	14.15 ± 0.22	15.78 ± 0.11
	5 (μg mL^*−*1^)	22.56 ± 0.12	15.5 ± 0.17	17.5 ± 0.207
Gentamycin	0.313 (μg mL^*−*1^)	9.25 ± 0.11	7.5 ± 0.27	NA
DMSO	0.313 (μg mL^*−*1^)	NA		9.05 ± 0.23

^NA^ Not active.

In experiments with the bacterial agent, the Ag–ZnO nanocomposite extract of Rumex Crispus yielded minimum inhibitory concentration (MIC) values between 0.313 and 0.125 mg/ml, whereas the range of 0.313–0.250 mg/ml was noted against the fungal strains. *Escherichia coli*, *Staphylococcus aureus*, and Candida albicans all had minimum lethal concentrations of 1.25, 0.625, and 2.5 g ml^−1^, respectively. All bacteria were highly susceptible to the least inhibitory dose of Ag–ZnO nanocomposite extract of Rumex Crispus at 0.313 mg/ml, according to the MIC values shown in Table 4.

### 3.6 Antioxidant activity

**Fig. 7:**
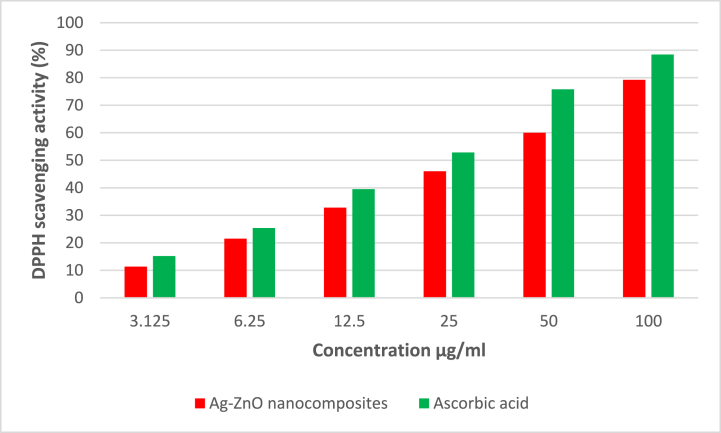
Antioxidant activity of synthetic Ag–ZnO nanocomposites against IC_50_ using DPPH free radical.

Fig. 7 displays the Ag–ZnO nanocomposites' antioxidant activity in combination with ascorbic acid and Rumex Crispus extract. The 1-1-diphenyl-2-picrylhydrazyl (DPPH) scavenging activity of Rumex Crispus extract and ascorbic acid at six different concentrations (3.125 to 100 g ml^−1^), ranging from 11.36 to 79.23% and 15.20 to 88.41% respectively, was the subject of the current investigation. Comparing the IC_50_ values of l-ascorbic acid (12.15 ± 0.025 g ml^−1^) and Rumex Crispus extract (29.31 ± 0.02 g ml^−1^), the DPPH experiment demonstrated the scavenging action of Ag–ZnO nanocomposites. Because of its high concentration of phenolics and flavonoids and other bioactive components in this plant, the Rumex Crispus extract nanocomposites were found to have antioxidant activity [40].

## 4 Conclusion

In this study, Ag–ZnO nanocomposites from Rumex Crispus extract were successfully produced for antibacterial and antioxidant properties using a straightforward hydrothermal technique. Using a Definite Screen Design, the impact of temperature, initial silver nitrate concentration, pH value, and reaction duration on the absorbance intensity of produced biosynthesis of the Ag–ZnO nanocomposites from Rumex Crispus extract was examined. The antibacterial and antioxidant activity of the produced Ag–ZnO nanocomposites was validated by FTIR, XRD, UV–vis, DLS, TGA, and DTA. DLS and XRD analysis revealed that biosynthesized Ag–ZnO nanocomposites were a polydispersity index value of 0.251 and were crystalline with average particle size from 23.44 nm with two distinct peaks with intensities of 97.8% and 2.2%. The gram-positive, gram-negative, and fungal strains all had minimum lethal concentrations of 1.25, 0.625, and 2.5 g ml^−1^, correspondingly. Thus, in this study, the Ag–ZnO nanocomposite extract of Rumex Crispus produced minimum inhibitory concentration (MIC) values between 0.313 and 0.125 mg/ml for the bacterial agent, whereas the range of 0.313–0.250 mg/ml was found against the fungal strains. Ag–ZnO nanocomposites have a scavenging effect on DPPH, and a Rumex Crispus extract's IC_50_ value is.29.31 μg ml^*–*^^1^. Additionally, it was discovered that the antibacterial properties of Ag–ZnO improved with higher reaction temperatures and silver nitrate salt dopant concentrations, suggesting that smaller composite material form at higher temperatures and Ag concentrations. The biosynthesized Ag–ZnO nanocomposites have potent antioxidant, antibacterial, and antifungal properties that make them an effective replacement for synthetic materials in the field of medical and biological applications for stated microorganism strains. Additionally, it is an eco-friendly way to make and use silver zinc oxide nanocomposites.

## Author contribution statement

Yigezu Mekonnen Bayisa, Tafere Aga Bullo, Ketema Beyecha Hundie, Desalegn Abdissa Akuma: Conceived and designed the experiments; Performed the experiments; Analyzed and interpreted the data; Contributed reagents, materials, analysis tools or data; Wrote the paper.

Defar Getahun Gizachew, Mohammed Seid Bultum: Analyzed and interpreted the data; Wrote the paper.

## Funding

This study did not receive any funding in any form.

## Data availability statement

The data that has been used is confidential.

## Ethical approval

Not applicable.

## Declaration of competing interest

The authors declare that they have no known competing financial interests or personal relationships that could have appeared to influence the work reported in this paper.
